# Training the Lung, Taming the NETs

**DOI:** 10.3389/fcimb.2026.1778635

**Published:** 2026-02-18

**Authors:** Piyush Baindara

**Affiliations:** 1 Animal Science Research Center, Division of Animal Sciences, University of Missouri, Columbia, MO, United States; 2 National Swine Testing Center, University of Missouri, Columbia, MO, United States

**Keywords:** host-directed therapies, infection, inflammation, NEtosis, trained-immunity

## Abstract

Respiratory infections remain a major global health threat, and recent epidemics have shown that treating the pathogen alone is not enough. In severe influenza, COVID-19, RSV, and bacterial pneumonia, lung failure often results less from microbial load and more from the host’s overactive immune response. Two key processes, neutrophil extracellular traps (NETs) and trained immunity, sit at the center of this shift toward host-focused intervention. Although both are innate defenses, they are usually discussed in isolation: NETs in the context of acute inflammation and thrombosis, and trained immunity in the context of vaccines, epigenetic reprogramming, and metabolic adaptation. Yet in the lung, these mechanisms function as interconnected elements of early defense. This editorial argues that effective therapies should no longer treat them as separate phenomena but instead co-target NET regulation and trained-immunity pathways as a unified, host-directed strategy to reduce immunopathology and improve outcomes in severe respiratory infections.

## NETs: from first responder to repeat offender

**Figure 1:**
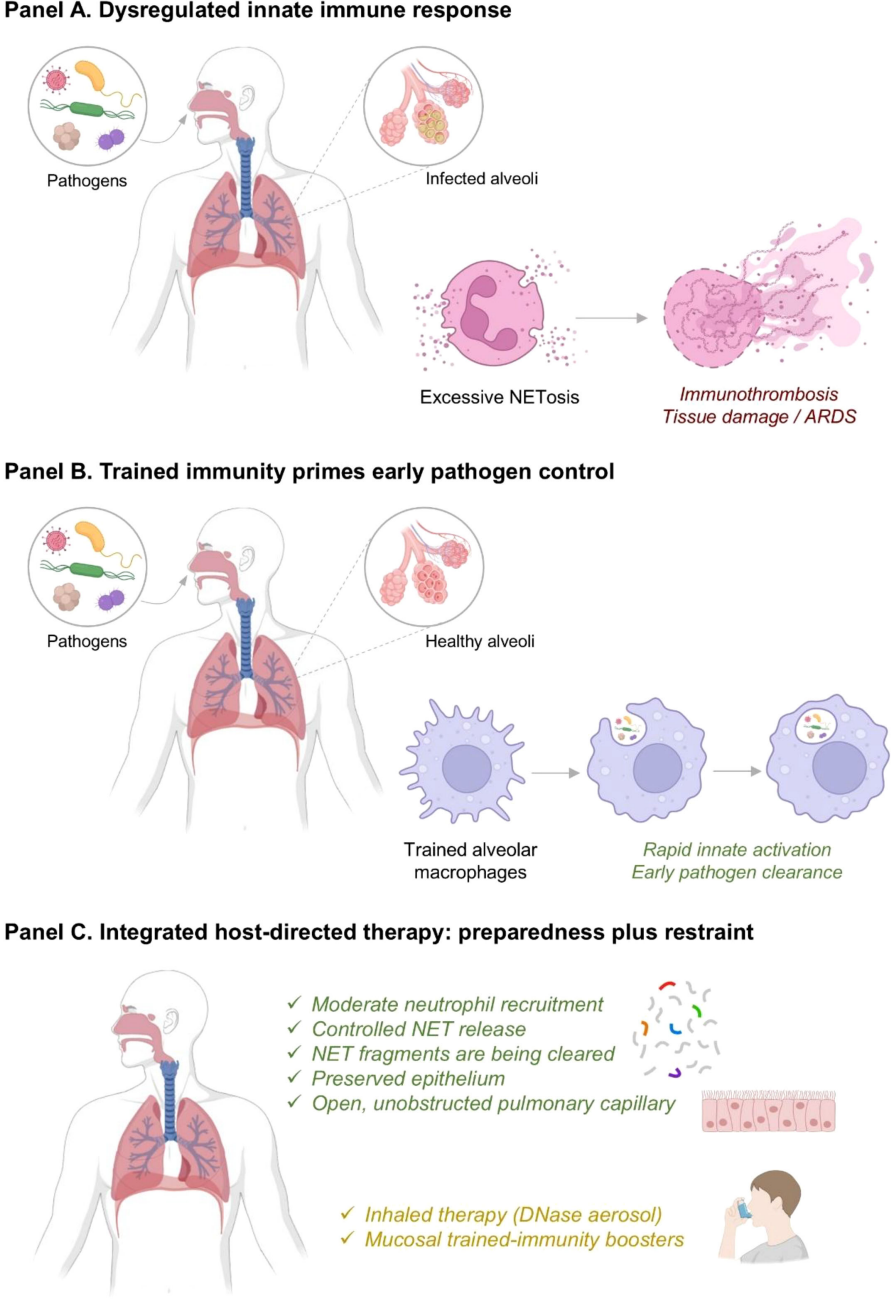
Innate immune dysregulation and NETs-based host-directed therapy in respiratory infection. **(A)** Pathogen-induced excessive NETosis promotes immunothrombosis, tissue injury, and ARDS. **(B)** Trained immunity enhances alveolar macrophage responsiveness, enabling rapid innate activation and early pathogen clearance. **(C)** Integrated host-directed strategies balance immune preparedness with restraint, supporting controlled NET release/clearance, epithelial preservation, and improved lung function, including inhaled DNase and mucosal trained-immunity boosters.

NETs were originally described as chromatin-based antimicrobial structures released by neutrophils to trap pathogens (Brinkmann et al., 2004). The lung is a central site for NET formation and trained immunity because of its continuous environmental exposure and rapid innate immune activation during infection (Netea et al., 2011). NETs were initially presented as a clear win for the host as webs of decondensed chromatin decorated with histones, proteases, and antimicrobial peptides (AMPs) that immobilize invading microbes within minutes. In principle, this is exactly what the lung needs when a high-dose viral or bacterial challenge reaches the distal airways. However, COVID-19 made it painfully obvious that NETs are a double-edged sword. Patients with severe COVID-19-related ARDS (acute respiratory distress syndrome) accumulate high levels of circulating and intra-pulmonary NETs that correlate with immunothrombosis, microvascular occlusion, and worse oxygenation. It has been shown that NETs were markedly elevated in patients with COVID-19 ARDS and directly contributed to a pro-thrombotic state (Middleton et al., 2020). NET markers in the serum have been successfully employed to identify patients with more severe COVID-19-associated respiratory disease symptoms, linking NET burden to clinical deterioration (Zuo et al., 2020). Moreover, dense NET infiltrates in the airways and parenchyma have been observed in the lung tissue of fatal COVID-19 patients (Radermecker et al., 2020). Notably, similar observations now extend to other forms of viral pneumonia and ARDS, where NETs have been observed in association with lung injury and organ dysfunction rather than simply with pathogen load (Ventura-Santana et al., 2022). The message is clear that the problem is not with the existence of NETs, but rather with their physiological boundaries. Completely blocking NET formation would be dangerous, as they are important for early containment of bacteria and fungi, especially at mucosal surfaces. However, when NETs production outstrips pathogen clearance, they become structural components of airway plugs, become fuel for immunothrombosis, and a persistent source of danger-associated molecular patterns. This is why the most promising therapies would be NETs-modulation, rather than NET-disruption. Importantly, DNase I, which degrades extracellular DNA, has been tested in patients with COVID-19 and other hyperinflammatory lung diseases, and early data suggested improved clearance of viscous secretions and trends toward better clinical outcomes when added to standard care (Earhart et al., 2020; Holliday et al., 2021). Moreover, in animal models of pneumonia and toxin-induced acute lung injury, NET degradation or pharmacologic inhibition of peptidylarginine deiminase 4 (PAD4), a key enzyme in chromatin decondensation, diminishes NET formation and attenuates lung damage (Zhou et al., 2024). Overall, the evidence is still emerging, but it already supports a conceptual shift. Instead of letting NETs run unchecked and then fighting downstream damage with steroids and supportive care, we should intervene earlier to keep NET levels in a protective, but non-destructive range (**Figure 1**). Although not unique to the lung, NETs and trained immunity are particularly prominent in respiratory tissues due to continuous microbial exposure and rapid innate immune activation.

## Trained immunity: a faster, smarter innate response

Trained immunity describes the long-term functional reprogramming of innate cells after certain infections or vaccines, leading to faster and often stronger responses to later challenges. This training is driven by epigenetic and metabolic changes in monocytes, macrophages, NK cells, and even tissue-resident populations such as alveolar macrophages (Domínguez-Andrés et al., 2023). Interestingly, early in the COVID-19 pandemic, it was proposed that trained immunity might be used to reduce susceptibility and severity of SARS-CoV-2 infection (Netea et al., 2020). Based on this idea, randomized trials such as ACTIVATE-2 showed that *Bacillus Calmette-Guérin* (BCG) revaccination in older adults reduced the incidence of new SARS-CoV-2 infections (Tsilika et al., 2022). Next, adenovirus-based COVID-19 vaccines have been shown to induce trained immunity (Netea and Joosten, 2023). Notably, β-glucans, yeast-derived polysaccharides, can train human monocytes and murine innate cells, enhancing antimicrobial function and boosting vaccine responses in experimental models of respiratory fungal infections (Moerings et al., 2021). More recently, it has been shown that with repeated or targeted exposures, the pulmonary environment itself can train alveolar macrophages and other innate cells, rewiring them to respond faster and more effectively to future challenges (Idiiatullina and Parker, 2025). Further, it is suggested that trained-immunity-based mucosal immunotherapies could offer broad protection against respiratory infections and effectively pre-arming the lung (Minute et al., 2025). Of course, trained immunity is a double-edged sword where the same mechanisms that strengthen early responses might drive chronic inflammation or maladaptive immune responses in COVID-19, including cardiovascular and autoimmune diseases (Tercan et al., 2021; Sviridov et al., 2025). However, in the context of acute respiratory infection, there is compelling experimental and early clinical evidence that appropriately tuned trained immunity can reduce viral load, lower the risk of secondary bacterial infections, and shorten the window of vulnerability (Schlüter et al., 2025). Altogether, these advances point toward trained immunity becoming a practical way to pre-arm the lung, offering rapid, broad protection that complements traditional vaccines (**Figure 1**).

## Why should NETs and trained immunity be considered together?

Right now, NETs and trained immunity are mostly discussed in different literatures. One set of papers asks how to stop neutrophils from destroying the lung, while another set asks how to prime innate cells so they respond more efficiently. In reality, they are two sides of the same early immune response. If a trained innate system eliminates or controls pathogens quickly, the subsequent wave of neutrophilic inflammation is smaller and shorter. Pathogens are cleared before they can spread widely, reducing the stimulus for sustained NET release. On the other hand, if training is absent or the host is immunologically naïve and vulnerable, pathogens can replicate unchecked, leading to a late surge of neutrophils and prolonged NETosis that damages tissue and promotes thrombosis. Based on this, NET overload is not simply an overreaction, but it is often a sign that early control failed. By the time clinicians are seeing massive NET deposition and immunothrombosis, the opportunity to shape the response upstream has largely been missed. Overall, this framing leads to a simple but important proposal that NET-modulating therapies and trained-immunity-inducing strategies should be developed and tested together, not separately. Overall, a combined strategy makes the most sense. Before or early in infection, trained-immunity inducers such as select mucosal vaccines, β-glucan formulations, or targeted innate-training adjuvants can boost the baseline readiness of innate cells in the respiratory tract (Minute et al., 2025). Next, as infection progresses, NET-modulating therapies like inhaled DNase to clear excessive NETs, or carefully dosed inhibitors of PAD4 or upstream signaling pathways, can keep neutrophil activity in a protective range rather than a damaging one (Espiritu and O’Sullivan, 2025). In simple terms, trained immunity helps prevent the need for an overwhelming neutrophil surge, while NET-modulation ensures that, if that surge does occur, it remains controlled instead of turning destructive.

## What stands in the way?

Combined treatment strategies, including NETs and trained immunity, seem promising; however, there are real challenges to implementing this vision. First of all, we are still lacking the promising routine biomarkers of NET burden and trained immunity that can be used in clinical decision-making. While circulating cell-free DNA, citrullinated histones, and MPO-DNA complexes have shown prognostic value in COVID-19, they are not yet part of standard care (Ondracek and Lang, 2021). Also, there is no simple trained immunity score for innate cells, even though epigenetic and metabolic signatures are increasingly well described. Second, NET-targeting therapies have not yet had the definitive trial that convinces clinicians. The emerging randomized data on inhaled dornase alfa in severe viral pneumonia are encouraging, but still in the early phase (Åkesson et al., 2025). Also, PAD4 inhibitors and other upstream NET-targeting agents are still early in development, with most data coming from animal studies rather than humans (Zhou et al., 2024). Third, trained immunity interventions face a credibility gap, such as BCG trials in adults have produced heterogeneous results, with some showing reduced respiratory infections and others showing no benefit or even slight increases in mild disease (Tsilika et al., 2022). Next, β-glucan preparations are diverse in source, purity, and route of administration, making it difficult to generalize across studies (Renke et al., 2022). Overall, these uncertainties argue for stronger, better-designed trials, not for abandoning the idea. A final challenge is that the relevant research communities still work in silos. NET biologists, pulmonologists, trained-immunity experts, and vaccine developers often operate in separate spaces, attend different meetings, and rarely design studies together. Because of this, potential interventions are tested one at a time instead of in the combinations that the underlying biology clearly suggests.

## A path forward

Beyond ARDS and COVID-19, dysregulated NET formation and altered innate immune responses have been implicated in cystic fibrosis and chronic obstructive lung diseases, highlighting the broader relevance of these mechanisms across pulmonary pathologies (Arts and Netea, 2016). However, specifically focusing on ARDS and COVID-19, none of these challenges is unbeatable, while they point to a clear research agenda. First, future ARDS and severe pneumonia studies should track both NET activity and markers of innate training, such as ex vivo cytokine responses and epigenetic changes in monocytes and alveolar macrophages, to understand how these pathways evolved. Second, early-phase clinical trials should adopt a staged design, testing trained-immunity boosters in high-risk individuals before or early in infection, and reserving inhaled DNase or other NET-modulating therapies for patients who progress to hypoxic pneumonia despite standard care. Third, emerging synthetic and bioengineered tools that fine-tune trained immunity in lung tissue without triggering chronic inflammation could be paired with equally precise NET-targeting agents. Finally, funding agencies should support interdisciplinary consortia that bring NET biologists, trained immunity researchers, and respiratory clinicians into the same projects. Notably, most strategies discussed here remain at the preclinical stage, with evidence derived largely from *in vitro* systems and animal models. Early clinical data, such as small randomized or observational studies of NET-modulating therapies, are emerging but remain limited in scale. Further, broad application in humans will require additional validation through well-powered clinical trials, improved biomarkers, and careful assessment of safety and durability over the coming years. Overall, in the era of increasing antimicrobial resistance and recurring respiratory pandemics, we cannot afford to treat each new pathogen as an isolated problem. NETs and trained immunity remind us that the host’s early innate response is a powerful, flexible system, one that can be steered toward protection or pathology. The question is not whether we should target the host, but how. In conclusion, it is plausible that high-risk patients receive a trained-immunity-based mucosal booster before viral season, followed by a precisely dosed NET-modulating inhaled therapy if they develop severe pneumonia. However, NETs are a protective defense mechanism, and disease arises from their dysregulation rather than their presence, underscoring the importance of modulation rather than complete inhibition. In summary, it just follows directly from what we already understand about the lung’s innate defenses. The real opportunity now is to design the interventions in a way that the immune system itself operates as a coordinated network where early preparedness and timely restraint work together to prevent damage and improve outcomes.

## Data Availability

The original contributions presented in the study are included in the article/supplementary material. Further inquiries can be directed to the corresponding author.
